# Evaluation of a new predictor of heart and left anterior descending artery dose in patients treated with adjuvant radiotherapy to the left breast

**DOI:** 10.1186/s13014-018-1069-z

**Published:** 2018-07-04

**Authors:** Lucas C. Mendez, Alexander V. Louie, Carolina Moreno, Matt Wronski, Andrew Warner, Eric Leung, Roberto Sakuraba, Juliana K. Helito, Ana Rezende, Icaro T. Carvalho, Eduardo Weltman

**Affiliations:** 1 Department of Radiation Oncology, Sunnybrook Health Sciences Centre, University of Toronto, 2075 Bayview Avenue, Toronto, ON M4N 3M5 Canada; 2 Department of Radiation Oncology, London Regional Cancer Program, Western University, London, ON Canada; 3 Division of Radiation Oncology, Hospital Israelita Albert Einstein, Sao Paulo, Brazil

**Keywords:** Breath-hold radiotherapy, Breast cancer, Prediction metrics

## Abstract

**Background:**

Heart-sparing techniques are time and resource intensive, although not all patients require the use of these strategies. This study evaluates the performance of different distance metrics in predicting the need for breath-hold radiotherapy in left-sided breast cancer patients receiving adjuvant radiotherapy.

**Methods:**

Fifty left-sided breast cancer patients treated with breast conserving surgery and adjuvant radiotherapy to the breast from a single institution were retrospectively studied. The left breast and organs at risk were contoured in accordance to guidelines and a plan with tangents was obtained using the free-breathing CT in supine position. Heart (mean heart dose (MHD), heart V25 Gy) and left anterior descending artery dosimetry were computed and compared against distance metrics under investigation (Contact Heart, 4th Arch and 5th Arch). Recursive partitioning analysis (RPA) was used to determine optimal cut-points for distance metrics for dosimetric end points. Receiver operating characteristic curves and Pearson correlation coefficients were used to evaluate the association between distance metrics and dosimetric endpoints. Univariable and multivariable logistic regression analysis was performed to identify significant predictors of dosimetric end points.

**Results:**

The mean MHD and heart V25 Gy were 2.3 Gy and 10.4 cm^3^, respectively. With tangents, constraints for MHD (< 1.7 Gy and V25 Gy < 10 cm^3^) were unattainable in 80% and 46% of patients, respectively. Optimal RPA thresholds included: Contact Heart (73 mm), 4th Arch (7 mm) and 5th Arch (41 mm). Of these, the 4th Arch had the highest overall accuracy, sensitivity, concordance index and correlation coefficient. All metrics were statistically significant predictors for MHD ≥ 1.7 Gy based on univariable logistic regression. Fifth Arch did not reach significance for heart V25 Gy ≥ 10 cm^3^. Fourth Arch was the only predictor to remain statistically significant after multivariable analysis.

**Conclusions:**

We propose a novel “4th Arch” metric as an accurate and practical tool to determine the need for breath-hold radiotherapy for left-sided breast cancer patients undergoing adjuvant radiotherapy with standard tangents. Further validation in an external cohort is necessary.

## Background

Adjuvant breast irradiation after breast-conserving surgery leads to similar oncological outcomes as mastectomy in patients with early breast cancer [1, 2] and improves survival in comparison to breast-conserving surgery alone [3]. Nevertheless, adjuvant RT, especially for left-sided breast cancer, also associates with cardiotoxicity and cardiovascular mortality. It is possible that radiotherapy cardio toxic effects may mitigate greater survival benefits associated with adjuvant radiotherapy in the long-term.

A landmark population-based study determined that adjuvant breast radiation was associated with a 1.7 fold increased risk of cardiac death when compared to patients treated with surgery alone [4]. This risk was notably higher in patients with left sided cancer. Additionally, a linear correlation between median heart dose and increased risk of major cardiac events was noted, strengthening the association between breast radiotherapy and heart damage [5]. With each additional 1 Gy of mean heart dose (MHD), there was a 7.4% incremental risk of major coronary events [5].

Different techniques have been proposed to mitigate radiation-induced heart damage. Breath-hold (BH) radiotherapy represents one option that holds promise as it can be employed in the context of a standard supine position and tangential delivery technique. In doing so, a dosimetric advantage can commonly be achieved with a deep inspiration BH due to the simultaneous effect of lung inflation and heart displacement out of the radiation field [6]. However, this technique does frequently require coaching, patient/staff coordination, apnea tolerance, as well as additional imaging and incremental time on the treatment unit. Moreover, not all patients may benefit dosimetrically from a BH technique, as some may possess a favorable thoracic geometry, inherently resulting in a small amount of cardiac radiation dose with standard tangential photon beams.

A study by Lee et al [7] evaluated different anatomic features that could be used to predict for the need of the BH technique in reducing cardiac dose in breast radiotherapy. Among the evaluated characteristics, this study found that the cranial-caudal distance of the heart in contact with the anterior chest wall (“Contact Heart”) measured in a free-breathing CT had the highest correlation with cardiac dose received.

We propose two novel and practical predictors based on anatomic landmarks that could be helpful in predicting the need for BH-radiotherapy. In this study, these metrics will be evaluated along with the metric previously suggested by Lee et al [7] in patients with left-sided breast cancers treated with breast-conserving surgery and being considered for adjuvant tangent radiotherapy. Our hypothesis was that these newer metrics could be used as a simpler and more accurate predictor of heart dose on the free-breathing scan and therefore predict the need for BH-radiotherapy.

## Methods

### Patient population

Fifty consecutive left-breast cancer patients treated with BH-radiotherapy between 2014 and 2015 were selected and evaluated in this planning study. All patients had histological proof of breast malignancy in core-biopsy. Nodal staging work-up was initially performed with clinical examination ± axilla ultrasound followed by sentinel nodal mapping during surgery.

The BH-radiotherapy institutional protocol was as follows: free-breathing and BH image sets were acquired by using Lightspeed computed tomography (CT) scan (GE Healthcare, Boston USA). CT images were acquired with patients in supine position on a 10 degrees breast board and with both arms elevated and abducted over 90 degrees. Slices of 3.5 mm thickness were obtained as per institutional protocol and a Real-time Position Management (RPM) device was used to obtain the breathing trace during simulation and treatment delivery.

### Contouring and plans

For the purpose of this study only free-breathing images were used to predict the need of BH technique by analyzing possible predictors. Breast, lungs and heart were contoured according to the RTOG guidelines [8] and left anterior descending coronary artery (LAD) was delineated following the heart atlas described by Feng et al [9]. The left breast was considered the clinical target volume (CTV) and the planning target volume (PTV) was generated by isotropically expanding the CTV contour by 7 mm. Radiation plans were obtained with tangential fields and 6 MV photons in Eclipse V.10 (Varian, Palo Alto USA). Segmented fields were also used when necessary to reduce dose heterogeneity. The dose prescription was 50 Gy and the institutional planning goals included a minimum PTV dose coverage of 47.5 Gy and a maximum point dose of 107% of the prescription dose. Heart and lung constraints followed the QUANTEC [10] definition. Dose to LAD was not taken into consideration during planning.

### Data collection

**Fig. 1:**
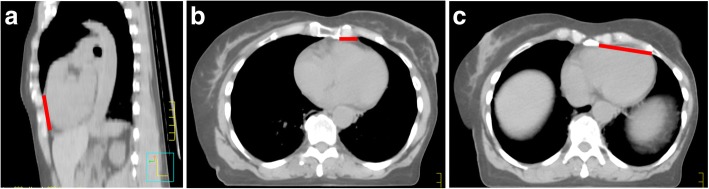
**a-c** Anatomically-based distance metrics for breath-hold radiotherapy showing (**a**) Contact Heart metric in the parasagittal axis and (**b**-**c**) 4th Arch and 5th Arch distance metrics, respectively acquired by a single measurement in the axial axis at the level in which the 4th or 5th costal arch connects with the sternum of adjacent costal cartilage

A set of anatomically-based distance metrics were obtained from each patient including the previously proposed “Contact Heart” metric [7] depicted in Fig. 1a showing the para-sagittal heart surface making contact with the anterior chest wall. This also included 2 additional metrics referred to as “4th arch” and “5th arch”, calculated by measuring the distance from the left sternal to the beginning of the lung parenchyma edges, at the 4th (Fig. 1b) and 5th (Fig. 1c) costal arch levels, respectively.

All plans were reviewed in quality assurance rounds after being approved by a radiation oncologist. Retrospectively, the primary end points of MHD, heart V25 Gy and maximum LAD point dose were collected and compared against the distance metrics previously described. MHD reflects the average dose received by the entire heart. This metric was previously correlated with cardiac events by Darby et al. [5] and the cut-off ≥1.7 Gy has been used as an indicator for breath-hold RT need [7]. Heart V25 Gy represents the volume of heart receiving 25 Gy or more. Gagliardi et al. [11] have correlated this dose with cardiac mortality and Wang et al. [12] indicated the need for BH-technique when more than 10 cm^3^ of the heart receives 25 Gy or more. As there is no definitive dosimetric parameter for LAD dose, we hypothesized that maximum LAD dose correlated with MHD and Heart V25Gy and the already known cut-points for these parameters.

### Statistical analysis

Descriptive statistics were generated for patient and treatment baseline characteristics for all patients (*n* = 50). Univariable recursive partitioning analysis (RPA) was performed based on MHD and heart V25 Gy BH end points to determine optimal cut-points separately for Contact Heart, 4th Arch and 5th Arch distance metrics and maximum LAD dose followed by rounding of cut-points to represent more clinically meaningful values. The association between distance metrics (Contact Heart, 4th Arch and 5th Arch) and BH end points (heart V25 Gy, MHD and maximum LAD dose) were evaluated using accuracy, sensitivity and specificity (for binary distance metrics) and concordance index and Pearson correlation coefficients (for continuous distance metrics). Pearson correlation coefficients were calculated using continuous BH endpoints. Receiver operating characteristic (ROC) curves were generated for MHD ≥ 1.7 Gy and heart V25 Gy ≥ 10 cm^3^ to further evaluate the association with Contact Heart, 4th and 5th Arch distance metrics. Univariable and multivariable logistic regression analysis were performed to identify significant predictors of BH end points. All statistical analysis was performed using SAS version 9.4 software (SAS institute, Cary NC) and the R language environment for statistical computing version 3.3.3 (open source, www.r-project.org), using two-sided statistical testing at the 0.05 significance level.

## Results

Fifty patients treated with breast-sparing surgery were included in this analysis. Forty-five patients underwent axilla investigation and in all cases nodes were negative. The mean age was 53.3 ± 12.0 years and the mean breast volume post-surgery was 630 ± 232 mL. Patient clinical and treatment characteristics are summarized in Table 1.

**Table 1:** Baseline patient, tumor and treatment characteristics for all patients (*n* = 50)

Characteristic	All Patients
Age – mean ± SD	53.3 ± 12.0
Breast Volume (cm^3^) – mean ± SD	630 ± 232
Tumor location in the breast (quadrants)^a^ – n (%)	
Superior	25 (66)
Inferior	7 (18)
Lateral	23 (61)
Medial	6 (16)
Tumor stage – n (%)	
T1	36 (74)
T2	7 (14)
T3	2 (4)
Tis	4 (8)
Node stage – n (%)	
N0	45 (90)
NX	5 (10)
Nodal investigation – n (%)	
None	5 (10)
Sentinel	34 (68)
Axillary	11 (22)
Mean Heart Dose (Gy) – mean ± SD	2.3 ± 0.8
Mean Heart Dose (Gy) – n (%)	
< 1.7 Gy	10 (20)
≥ 1.7 Gy	40 (80)
Heart Volume (cm^3^) – mean ± SD	564 ± 87.2
Heart V25 Gy (cm^3^) – mean ± SD	10.4 ± 9.7
Heart V25 Gy (cm^3^) – n (%)	
< 10 cm^3^	27 (54)
≥ 10 cm^3^	23 (46)
Maximum LAD Dose (Gy) – mean ± SD	37.5 ± 15.7

^a^Column totals exceed 100% as some tumors were located in multiple breast quadrants;

*Tis* Tumour in situ, *LAD* Left anterior descending coronary artery, *N* Number

The mean MHD was 2.3 ± 0.8 Gy and 40 patients were found to have MHD ≥ 1.7 Gy. Mean Heart V25 Gy was 10.4 ± 9.7 cm^3^ and 23 patients had V25 Gy ≥ 10 cm^3^. Maximum LAD dose of ≥28 Gy was considered as an optimal cut-point constraint for this structure, based on MHD and V25 Gy constraints using an RPA approach and incorporated as an additional BH end point. Thirty-seven patients were found to have LAD ≥28 Gy.

**Fig. 2:**
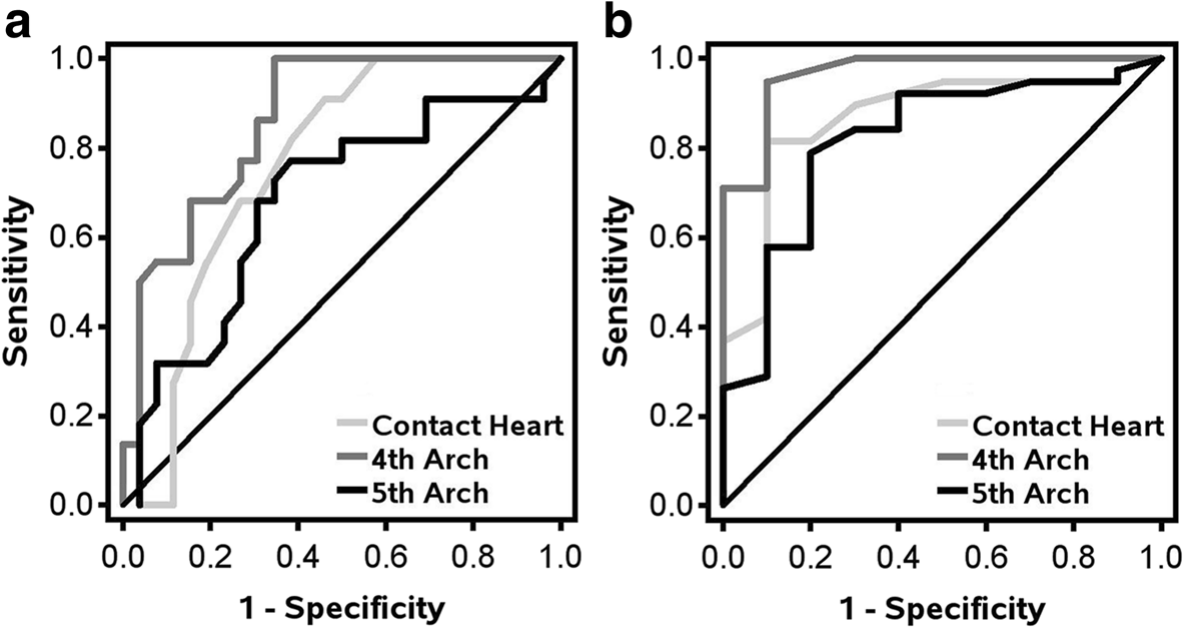
**a-b** Receiver operating characteristic curves for(**a**) heart V25 Gy ≥ 10 cm3 (ContactHeart AUC: 0.76; 4th Arch AUC: 0.86; 5th Arch AUC: 0.68) and (**b**) mean heart dose ≥1.7 Gy (ContactHeart AUC: 0.87; 4th Arch AUC: 0.97; 5th Arch AUC: 0.82) for Contact Heart, 4th Arch and 5th Arch distance metrics

The association between distance metrics using RPA derived cut-points and BH end points are summarized Table 2. For comparison, Contact Heart was additionally shown using the previously reported cut-point of 50 mm [7]. RPA identified optimal cut-points of 73 mm for Contact Heart, 7 mm for 4th Arch and 41 mm for 5th Arch. Fourth Arch had the highest overall accuracy (range: 68.0–94.0), sensitivity (range: 94.6–100), concordance index (range: 0.86–0.97) and Pearson correlation coefficient (range: 0.55–0.61) across all BH end points. Due to the low specificity of Contact Heart 50 mm in comparison to 73 mm for all BH end points, 50 mm was excluded from further analysis. ROC curves for MHD ≥ 1.7 Gy and heart V25 Gy ≥ 10 cm^3^ comparing distance metrics are shown in Fig. 2.

**Table 2:** Association between distance metrics using recursive partitioning analysis derived cut-points and each breath-hold end point using accuracy, sensitivity and specificity (binary distance metrics) and concordance index and Pearson correlation coefficients (continuous distance metrics). Pearson correlation coefficients calculated using continuous breath-hold end points

Breath-Hold End Points:		Heart V25 Gy ≥ 10 cm^3^					Mean Heart Dose ≥1.7 Gy					Maximum LAD Dose ≥28 Gy				
Variables (mm)	Mean ± SD n (%)	Acc	Sens	Spec	C	PCC	Acc	Sens	Spec	C	PCC	Acc	Sens	Spec	C	PCC
Contact Heart	78.3 ± 22.8	–	–	–	0.76	0.31^b^	–	–	–	0.87	0.32^b^	–	–	–	0.84	0.49^b^
< 73 mm ≥ 73 mm	18 (36) 32 (64)	70.0	87.0	55.6	–	–	80.0	77.5	90.0	–	–	82.0	81.1	84.6	–	–
< 50 mm^a^ ≥ 50 mm^a^	5 (10) 45 (90)	56.0	100	18.5	–	–	82.0	95.0	30.0	–	–	80.0	97.3	30.8	–	–
4th Arch	32.8 ± 25.5	–	–	–	0.86	0.57^b^	–	–	–	0.97	0.61^b^	–	–	–	0.91	0.55^b^
< 7 mm ≥ 7 mm	11 (22) 39 (78)	68.0	100	40.7	–	–	94.0	95.0	90.0	–	–	88.0	94.6	69.2	–	–
5th Arch	58.5 ± 23.4	–	–	–	0.68	0.12	–	–	–	0.82	0.16	–	–	–	0.76	0.40^b^
< 41 mm ≥ 41 mm	9 (19) 39 (81)	54.0	90.9	26.9	–	–	82.0	92.1	60.0	–	–	78.0	91.7	50.0	–	–

*LAD* Left anterior descending coronary artery, *Acc* Accuracy, *Sens* Sensitivity, *Spec* Specificity, *C* Concordance index, *PCC* Pearson correlation coefficient;

^a^50 mm cut-point identified previously and for comparison purposes only (not identified from recursive partitioning analysis)

^b^*p* <  0.05

Univariable logistic regression analysis is shown in Table 3 and demonstrated that Contact Heart (*p* = 0.002), 4th Arch (*p* <  0.001) and 5th Arch (*p* = 0.001) were each statistically significant predictors for MHD ≥ 1.7 Gy. Fifth Arch did not reach significance for Heart V25 Gy ≥ 10 cm^3^ (*p* = 0.131). Following multivariable logistic regression analysis, only 4th Arch remained statistically significant for both MHD (*p* <  0.001) and Heart V25 Gy (*p* <  0.001).

**Table 3:** Univariable logistic regression models of distance metrics predictive of breath-hold and dosimetric end points (heart V25Gy ≥ 10 cm^3^, mean heart dose ≥1.7 Gy and maximum left anterior descending coronary artery dose ≥28 Gy)

Dependent Variables:	Heart V25Gy ≥ 10cm^3^		Mean Heart Dose ≥ 1.7Gy		Maximum LAD Dose ≥ 28Gy	
Variables:	OR (95% CI)	*p*-value	OR (95% CI)	*p*-value	OR (95% CI)	*p*-value
Contact Heart Distance ≥ 73 mm (vs. < 73 mm)	8.33 (1.99, 34.86)	0.004	31.00 (3.45, 278)	0.002	23.57 (4.24, 131)	< 0.001
4th Arch Distance ≥ 7 mm (vs. < 7 mm)	20.20 (3.86, ∞)	**< 0.001**	171 (13.93, ∞)	**< 0.001**	39.38 (6.20, 250)	**< 0.001**
5th Arch Distance ≥ 41 mm (vs. < 41 mm)	3.68 (0.68, 20.01)	0.131	17.50 (3.10, 98.65)	**0.001**	11.00 (2.14, 56.47)	**0.004**

*OR* Odds ratio, *CI* Confidence interval, *LAD* Left anterior descending coronary artery

*P*-values < 0.05 shown in BOLD

## Discussion

Breath-hold radiotherapy has been shown to significantly reduce cardiac dose [6], which may mitigate long-term cardiovascular toxicity. Nevertheless, BH treatment is laborious as well as time and resource-intensive [12]. Furthermore, some left-breast cancer patients may not benefit substantially from this technique due to favorable anatomy. Therefore, the utility of a simple metric to predict the necessity of BH-radiotherapy before the process of coaching and planning takes place would help improve efficiency within a busy clinical environment.

Previously, Lee et al. investigated predictors of heart dose and observed that the para-sagittal heart surface contact distance with the anterior chest wall (“Contact Heart”) demonstrated good accuracy [7]. Despite its reasonable prediction capacity, this metric is not very practical for routine use because it requires all CT slices in which the heart is in contact with the thoracic wall to be counted and then multiplied by the slice thickness. Of note, due to partial volume effects, it is not clear if this metric would maintain similar accuracy and reproducibility in protocols that use thicker CT slices than that used by Lee et al.

In this study, we proposed two alternative predictors and would contend that they are more practical than previously proposed metrics. Both involve a simple linear measurement from the left edge of the sternum to the anterior portion of the left lung either to the 4th (“4th Arch”) or 5th (“5th Arch”) costal arch level, respectively (Fig. 1b-c). These metrics are less likely to be dependent on the CT-scan slice thickness, given the measurement is performed in the axial plane and not in the cranial-caudal direction.

A useful predictor should be simple yet also accurate. This study compared and evaluated 3 distance metrics using a variety of accuracy and prediction based techniques. Fifty patients with left-side breast cancer treated with adjuvant radiotherapy after breast-sparing surgery were randomly selected for inclusion in the study. Heart dosimetric parameters were used to quantify the need for the BH-technique. After comprehensive analysis, the 4th Arch metric was found to have the highest overall predictive accuracy and sensitivity for MHD and Heart V25 Gy as well as maximum LAD dose. This metric was also associated with the largest concordance index and correlation coefficient when compared to the 5th Arch and Contact Heart metrics for all BH end points. Moreover, the 4th Arch metric was observed to be the strongest predictor of each BH end point from univariable logistic regression and remained the only statistically significant predictor following multivariable analysis.

MHD ≥ 1.7 Gy and Heart V25 ≥ 10 cm^3^ were considered in this study as hard-constraints, as these dosimetric values were correlated with cardiovascular toxicity in previous publications [5, 11, 12]. These parameters were used to define the optimal LAD cut point dose (≥ 28Gy) through RPA techniques and therefore used in this study as an additional exploratory BH end point. Similarly to previous results, the 4th Arch metric was also shown to have the strongest correlation with LAD dose.

Recent publication by Rahimy et al. underscores the continuous value of breast tangents in the modern era [13]. In this analysis, authors have shown that inverse-planning treatments based on 2-gantry angles outperform more elaborate plans with multiple gantry angles in regard to MHD, which highlights the continued relevance of this classical technique in current times and the importance of the topic studied here. In the post-breast reconstruction setting, Lancellotta et al. have also seen lower MHD with 3D-based tangencial fields in comparison to more complex techniques [14].

This planning study needs to be considered in the context of its limitations. First, although patients were randomly sampled from an institutional database, the generalizability of the diagnostic performance of our proposed metric needs to be validated with an independent dataset. Second, the imaging protocol, in particular the slice thickness, was different from the protocol reported by Lee et al. [7]. It is possible that this modification could compromise the performance seen with the “Contact Heart” metric, although in that case, the robustness of the metric in other imaging protocols would be under question. Third, window-leveling in which the 4th Arch is measured may be a drawback associated with this metric and this feature was not assessed in this study. Finally, future evaluation of the inter-measurement variability among healthcare professionals involved in breast radiotherapy planning is necessary for a complete assessment of this metric’s value.

## Conclusions

In conclusion, we propose that the 4th Arch metric is a simple and practical method that has demonstrated a strong correlation with heart and LAD dosimetry in left sided-breast cancer patients treated with standard tangential radiation therapy. Prospective and independent validation of this metric should be considered in the future for quantification of the need for heart sparing using BH or other techniques before its routine employment.

## Abbreviations

BH: Breath-hold
CT: Computed tomography
CTV: Clinical target volume
LAD: Left anterior descending coronary artery
MHD: Mean heart dose
PTV: Planning target volume
RPA: Recursive partitioning analysis
RT: Radiotherapy
